# Cross-tissue immune profiling of APOE ε4 reveals early dysregulation in Alzheimer’s disease

**DOI:** 10.21203/rs.3.rs-7089423/v1

**Published:** 2025-07-15

**Authors:** Caitlin Finney, Artur Shvetcov, Shannon Thomson, Mark Graham, Brittany Hauger, Jessica Keller, Christine Smoyer, Sarah Tague, Ann-Na Cho, Farhad Imam, Varsha Krish, Global Neurodegeneration Proteomics Consortium Global Neurodegeneration Proteomics Consortium, Matthew Taylor, Jonathan Mahnken, Debra Sullivan, Joanne Reed, Jeffrey Burns, Chad Slawson, Russell Swerdlow, Heather Wilkins

**Affiliations:** Westmead Institute for Medical Research; Westmead Institute for Medical Research; Westmead Institute for Medical Research; Children’s Medical Research Institute; University of Kansas Alzheimer’s Disease Research Center; University of Kansas Alzheimer’s Disease Research Center; University of Kansas Medical Center; University of Kansas Medical Center; University of Sydney; Gates Ventures; Gates Ventures; Gates Ventures; University of Kansas Alzheimer’s Disease Research Center; University of Kansas Alzheimer’s Disease Research Center; University of Kansas Alzheimer’s Disease Research Center; Westmead Institute for Medical Research; Univesrity of Kansas Medical Center; University of Kansas Alzheimer’s Disease Research Center; University of Kansas; University of Kansas Alzheimer’s Disease Research Center, University of Kansas Medical Center, Kansas City

## Abstract

APOE ε4 is the strongest genetic risk factor for late-onset Alzheimer’s disease (AD), but its contribution to disease pathogenesis remains incompletely understood. Here, we integrate proteomic profiling of plasma, cerebrospinal fluid (CSF), and brain tissue from over 10,000 individuals to define the immune phenotype associated with APOE ε4. We identify a conserved, allele dose-dependent pro-inflammatory immune signature across peripheral and central tissues independent of AD diagnosis. This signature is enriched in adaptive immune cells and white matter-resident glial and vascular cells. It also emerges in patient-derived cortical organoids prior to amyloid-β and tau pathology, supporting a causal, genotype-driven mechanism. Cross-tissue comparisons reveal shared innate and antiviral responses alongside tissue-specific immune signaling. Notably, a 12-week medical ketogenic diet partially reversed the APOE ε4 immune signature. These findings position immune dysregulation as an early and tractable driver of AD risk in APOE ε4 carriers with direct implications for targeted prevention strategies.

The functional consequences of the largest genetic risk factor for late onset Alzheimer’s disease (AD), the ε4 variant of the apolipoprotein E (*APOE*) gene, and how they drive AD pathogenesis remain poorly understood. Recent accumulating evidence suggests that *APOE* ε4 exerts broad modulatory effects on both innate and adaptive immunity across peripheral tissues and the central nervous system. *APOE* ε4 is associated with a shift toward a heightened pro-inflammatory state ^1,2^. This is further evidenced by peripherally-derived immune cells showing exaggerated responses to innate immune stimulation ^3,4^, altered antigen presentation ^5^, disrupted T-cell homeostasis ^6^, and signs of accelerated immune aging ^7^. In the brain, *APOE* ε4 carriage is linked to pre-activated microglial phenotypes ^8^, impaired lipid and autophagic homeostasis ^9,10^, and amplified responses to amyloid-β pathology ^10,11^. Together, these findings highlight a genotype-dependent dysregulation of immune homeostasis in *APOE* ε4 carriers. However, several key questions remain, including whether immune changes represent innate genetic effects or secondary responses to disease, how these signatures evolve temporally with progression to AD, which aspects are systemic, central, or peripheral in origin, and whether they are modifiable. Resolving these questions is essential to understanding *APOE* ε4-driven mechanisms of neurodegeneration and developing precision biomarkers and early intervention strategies for AD.

To address how *APOE* ε4 influences immune function across tissues and time, we systematically profiled the plasma, CSF, and brain proteomes of APOE ε4 carriers and non-carriers with and without AD (Fig. 1a). We identified a conserved, allele dose-dependent pro-inflammatory immune signature spanning both the periphery and central nervous system (CNS), independent of disease. This signature was recapitulated in patient-derived cortical organoids and emerged prior to the development of amyloid-β and tau pathology, indicating that *APOE* ε4-driven immune changes are early, intrinsic, and may initiate downstream neurodegenerative processes. Cross-tissue comparisons revealed that many *APOE* ε4-associated pathways were systemic, reflecting broad innate and adaptive immune activation. Others were tissue-specific and localized to the CNS or periphery. Importantly, short-term treatment with an anti-inflammatory medical ketogenic diet in *APOE* ε4 carriers with AD partially reprogrammed this immune phenotype, reducing pro-inflammatory signaling and promoting regulatory and tissue-supportive immune functions. Together, these findings demonstrate that *APOE* ε4 confers a systemic but locally modulated pro-inflammatory immune phenotype that emerges early, evolves with disease progression, and may promote AD pathology. This provides new insight into *APOE* ε4-driven mechanisms of neurodegeneration and highlights the potential of this immune state to be therapeutically modulated.

## Results

### *APOE* ε4 carriers have a distinct pro-inflammatory immune phenotype in the plasma and cerebrospinal fluid

**Figure 1:**
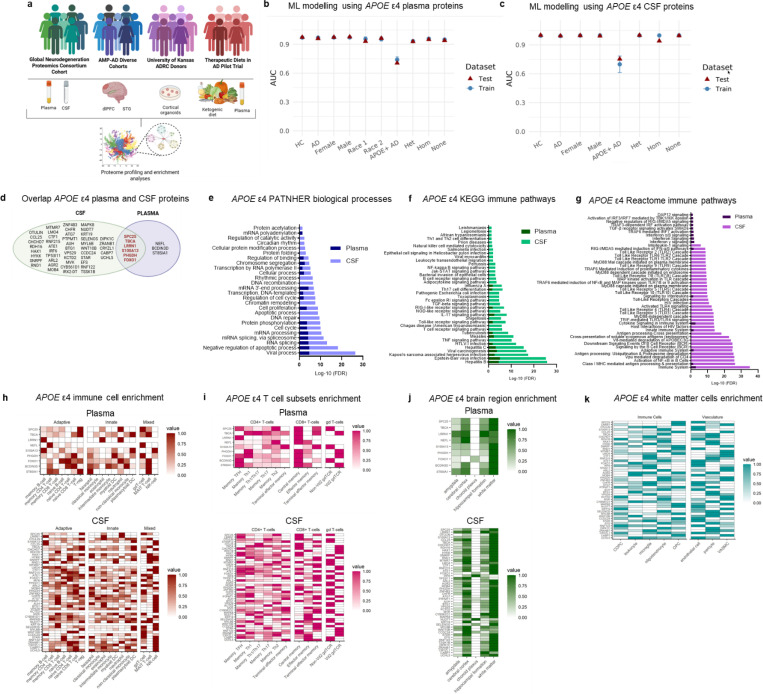
Study design and characterization of the plasma and cerebrospinal fluid proteome of *APOE* ε4 carriers and non-carriers with and without Alzheimer’s disease. (**a**) Design of the current study using plasma and cerebrospinal (CSF) proteomic data from the Global Neurodegeneration Proteomics Consortium cohort, brain proteomic data from the dorsolateral prefrontal cortex (dlPFC) and superior temporal gyrus (STG) of donors from the Accelerating Medicines Partnership in Alzheimer’s Disease Diverse Cohorts study, cortical organoids generated from donors from the University of Kansas Alzheimer’s Disease Research Center cohort, and plasma proteomic data from the Therapeutic Diets in AD pilot clinical trial of 12 week treatment with an anti-inflammatory medical ketogenic diet. (**b**) Performance metrics of machine learning models using *APOE* ε4 plasma proteins as features. The graph shows mean area under the curve (AUC) + SD for both testing and training datasets across five folds repeated 10 times. Models were trained and validated on a 70% training dataset and tested using a 30% withheld testing dataset. Race 1 represents Black or African American participants and Race 2 represents American Indian or Alaskan Native participants. (**c**) Performance metrics of machine learning models using the 51 *APOE* ε4 CSF proteins as features. The graph shows mean area under the curve (AUC) + SD for both testing and training datasets across five folds repeated 10 times. Models were trained and validated on a 70% training dataset and tested using a 30% withheld testing dataset. (**d**) Venn diagram showing the overlap between identified CSF and plasma *APOE* ε4 proteins. (**e**) Significantly (FDR < 0.05) enriched PANTHER biological processes *APOE* ε4 plasma and CSF proteins, with viral processes as the top enriched pathway across both. (**f**) Significantly (FDR < 0.05) enriched KEGG immune pathways for *APOE* ε4 plasma and CSF proteins. Enriched pathways included those involved in pro-inflammatory, cytokine, infection, and both adaptive and innate immune function. (**g**) Significantly (FDR < 0.05) enriched Reactome immune pathways for *APOE* ε4 plasma and CSF proteins. Enriched pathways mirror those found with KEGG, with enrichment for pro-inflammatory, cytokine, and broad adaptive and innate immune functions. (**h**) Enrichment of *APOE* ε4 plasma and CSF proteins across distinct immune cell subsets. Plasma proteins were especially enriched across neutrophils, basophils, and T cells. CSF proteins were more enriched in T cells and NK cells. (**i**) Given that we found high enrichment for T cells, broadly, we did a further enrichment for distinct T-cell subsets. *APOE* ε4 plasma proteins were the most enriched for central memory CD8^+^ T cells and CD4^+^ memory TFH cells. CSF *APOE* ε4 proteins were also enriched in CD4^+^ memory TFH cells as well as CD4^+^ memory Th1, CD8^+^ terminal effector memory, and non-Vδ2 γδ T cells. (**j**) Enrichment of *APOE* ε4 plasma and CSF proteins across commonly affected brain regions in AD, with the highest enrichment for white matter. (**k**) Further enrichment of *APOE* ε4 CSF proteins across white matter cell subtypes. Microglia and oligodendrocytes were the most enriched immune cells and endothelial and vascular cells were the most enriched vascular cells. Enrichments are based on single cell RNA-sequencing data from the Human Protein Atlas ^12–14^ and plots show mix-max scaling of protein-coding transcripts per million for each identified *APOE* ε4 plasma protein. Abbreviations: AD: Alzheimer’s disease; COPC: committed oligodendrocyte precursor cell; CSF: cerebrospinal fluid; DC: dendritic cell; dlPFC: dorsolateral prefrontal cortex; Het: heterozygous; Hom: homozygous; NI: non-impaired control; OPC: oligodendrocyte precursor cell; STG: superior temporal gyrus; TFH: T follicular helper T cells; VASMC: vascular-associated smooth muscle cells.

We profiled 6,340 plasma proteins of *n* = 2,929 AD and *n* = 6,099 non-impaired controls from the Global Neurodegeneration Proteomics Consortium (GNPC) using the SomaScan 7k assay (Supplementary Table 1). Nine *APOE* ε4-associated plasma proteins were identified (mutual information > 0.1), which clustered by carrier status and allele dose (Supplementary Table 2; Extended Data Fig. 1a-c). Machine learning using classification and regression trees (CART) showed these proteins robustly classified *APOE* ε4 carriers versus non-carriers (AUC > 0.91 across subgroups; Extended Data Table 1; Fig. 1b), independent of AD status, sex, or race. No plasma proteins differentiated AD *APOE* ε4 carriers from non-impaired carriers (mutual information < 0.01; AUC = 0.70), confirming an AD-independent signature. Multi-class CART further distinguished heterozygous from homozygous carriers (AUC > 0.93).

We then analyzed 6,340 CSF proteins from *n* = 526 AD and *n* = 573 non-impaired controls (Supplementary Table 1), identifying 51 *APOE* ε4-associated proteins (mutual information > 0.1) that similarly clustered by carrier status and allele dose (Supplementary Table 3; Extended Data Fig. 1d-f). CART models accurately classified *APOE* ε4 carriers (AUC = 0.99), again independent of AD status or sex, and distinguished between heterozygous and homozygous individuals (AUC > 0.94; Extended Data Table 2; Fig. 1c). As in plasma, no CSF proteins distinguished AD from non-impaired *APOE* ε4 carriers (mutual information < 0.01; AUC = 0.75), further supporting an AD-independent immune signature.

Six of the nine plasma proteins overlapped with CSF *APOE* ε4 proteins (Fig. 1d). Pathway analyses revealed significant enrichment for viral processes (FDR < 0.05) in both plasma and CSF (PANTHER; Fig. 1e; Supplementary Table 4). KEGG and Reactome analyses confirmed enrichment for immune pathways including interferon, interleukin, Th17, TGF-β signaling, Epstein-Barr virus and hepatitis (Fig. 1e-f; Supplementary Table 4). CSF-specific pathways included toll-like receptor signaling and NK/B cell pathways, while plasma-specific enrichments were dominated by interferon signaling (Fig. 1e-f; Supplementary Table 4). Cell-type enrichment using Human Protein Atlas single-cell RNA-sequencing data ^12,13^, showed plasma *APOE* ε4 proteins were enriched in innate immune cells (neutrophils, basophils) and T cells, particularly central memory CD8^+^ and CD4^+^ TFH subsets (Fig. 1h-i). CSF proteins were enriched in NK cells and adaptive T-cell subsets, including CD4^+^ Th1 and CD8^+^ terminal effector memory cells (Fig. 1i). Both plasma and CSF *APOE* ε4 proteins were preferentially expressed in white matter, with further enrichment in microglia, oligodendrocytes, and vascular-associated cells in CSF (Fig. 1j-k).

Together, these findings define a robust, AD-independent, allele dose-sensitive immune signature in *APOE* ε4 carriers that spans the plasma and CNS and is enriched in adaptive immune and white matter-resident cell types.

### The *APOE* ε4 phenotype extends into the brain, with the superior temporal gyrus especially affected

**Figure 2:**
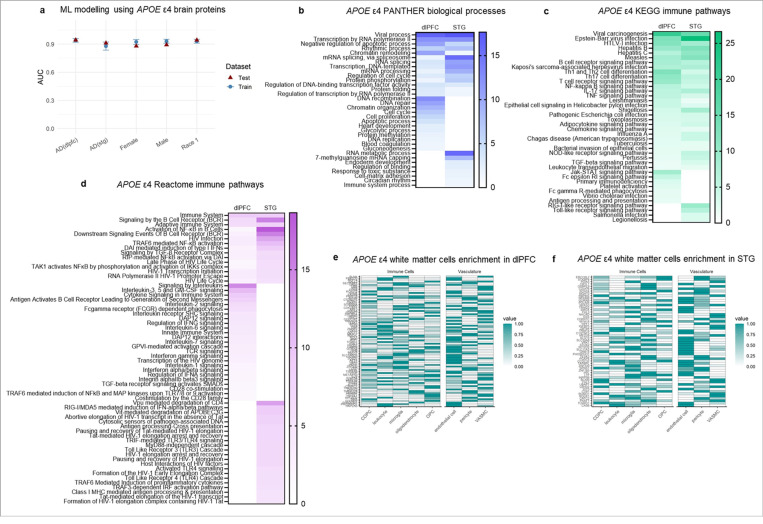
Characterization of the dorsolateral prefrontal cortex and superior temporal gyrus brain proteomes of *APOE* ε4 carriers and non-carriers with and without Alzheimer’s disease. (**a**) Performance metrics of machine learning models using the *APOE* ε4 dorsolateral prefrontal cortex (dlPFC) or superior temporal gyrus (STG) proteins as features. The graph shows mean area under the curve (AUC) + SD for both testing and training datasets across five folds repeated 10 times. Models were trained and validated on a 70% training dataset and tested using a 30% withheld testing dataset. Race 1 refers to Black or African American donors. (**b**) Significantly (FDR < 0.05) enriched PANTHER biological processes for the *APOE* ε4 dlPFC and STG proteins. The top enriched biological process across both regions was viral processes (FDR = 3.95E-17 and 2.18E-18, respectively). The scale bar represents −1og10(FDR). (**c**) Significantly (FDR < 0.05) enriched KEGG immune pathways for the *APOE* ε4 dlPFC and STG proteins. Enriched pathways included those involved in pro-inflammatory, cytokine, infection, and both adaptive and innate immune function. The scale bar represents −1og10(FDR). (**d**) Significantly (FDR < 0.05) enriched Reactome immune pathways for the *APOE* ε4 dlPFC and STG proteins. Enriched pathways mirror those found with KEGG, with enrichment for pro-inflammatory, cytokine, and broad adaptive and innate immune functions. Enrichment for these pathways was more significant in the STG than the dlPFC. The scale bar represents −1og10(FDR). (**e,f**) Given our previous findings in the plasma and CSF showing *APOE* ε4 proteins were enriched in white matter, we performed an enrichment analysis for white matter cell subtypes. (**e**) dlPFC *APOE* ε4 proteins were enriched for microglia as well as endothelial cells. (**f**) STG *APOE* ε4 proteins were similarly enriched for microglia and endothelial cells. Enrichments are based on single cell RNA-sequencing data from the Human Protein Atlas ^14^ and plots show mix-max scaling of protein-coding transcripts per million for each identified *APOE* ε4 dlPFC or STG protein. Abbreviations: AD: Alzheimer’s disease; COPC: committed oligodendrocyte precursor cell; CSF: cerebrospinal fluid; dlPFC: dorsolateral prefrontal cortex; NI: non-impaired control; OPC: oligodendrocyte precursor cell; STG: superior temporal gyrus; VASMC: vascular-associated smooth muscle cells.

To assess whether the *APOE* ε4-associated immune phenotype extended into the brain, we analysed TMT-based proteomic data from the Accelerating Medicine Partnerships in AD (AMP-AD) Diverse Cohorts study in the dorsolateral prefrontal cortex (dlPFC; *n* = 530 AD, *n* = 190 non-impaired controls) and superior temporal gyrus (STG; *n* = 161 AD, *n* = 49 non-impaired controls; Supplementary Table 5). We identified 85 *APOE* ε4-associated proteins in dlPFC and 56 in STG of non-impaired controls (mutual information > 0.1; Supplementary Table 6), with only two proteins overlapping across regions, GORAB and PHOSPHO1, suggesting region-specific effects of *APOE* ε4. Coverage differences between SomaScan and TMT precluded direct plasma/CSF comparisons. Random forest modelling showed that *APOE* ε4 brain proteins reliably classified *APOE* ε4 carriers versus non-carriers with AD (dlPFC AUC = 0.94; STG AUC = 0.91), independent of sex and race for dlPFC (AUC > 0.88; Extended Data Table 3; Fig. 2a). Insufficient sample size precluded subgroup analysis in STG.

Consistent with plasma and CSF findings, viral processes were the most significantly enriched *APOE* ε4 associated biological processes (PANTHER; Fig. 2b; Supplementary Table 7). KEGG analyses revealed significant enrichment for pro-inflammatory, infection-related, and adaptive immune pathways. There were also region-specific pathways including Fc receptor and platelet activation in dlPFC, and TLR and RLR signaling in STG (Fig. 2c; Supplementary Table 7). Reactome pathways overlapped across regions for adaptive immune, B cell receptor, and HIV-related pathways. dlPFC-specific pathways reflected immune cell activation and cytokine regulation whereas STG pathways were more focused on pathogen sensing and antiviral defense (Fig. 2d; Supplementary Table 7). Across both regions, *APOE* ε4 brain proteins were preferentially enriched in microglia and endothelial cells (Fig. 2e).

These findings identify distinct, region-specific *APOE* ε4 brain protein signatures, independent of sex and race, that mirror the viral, pro-inflammatory, and adaptive immune pathway enrichment seen in plasma and CSF. The regional differences suggest nuanced immune and pathogen-sensing functions in *APOE* ε4 brains, with microglia and cerebrovascular endothelial enrichment implicating white matter and vascular immune processes in the *APOE* ε4 brain phenotype.

### Stem cell-derived cortical organoids show the *APOE* ε4 phenotype before Alzheimer’s pathology onset

Across two clinical cohorts, we demonstrated that *APOE* ε4 carriers exhibit a pro-inflammatory immune phenotype both peripherally and centrally. We previously showed that this phenotype is not correlated with hallmark AD pathologies including amyloid-β, tau, gliosis, or angiopathy ^2^. However, since these clinical samples are derived from symptomatic individuals or asymptomatic individuals who may harbor subclinical AD pathology, it remains unclear whether this *APOE* ε4 immune phenotype precedes or follows the development of AD pathology, a critical question for guiding diagnosis and treatment in *APOE* ε4 carriers.

**Figure 3:**
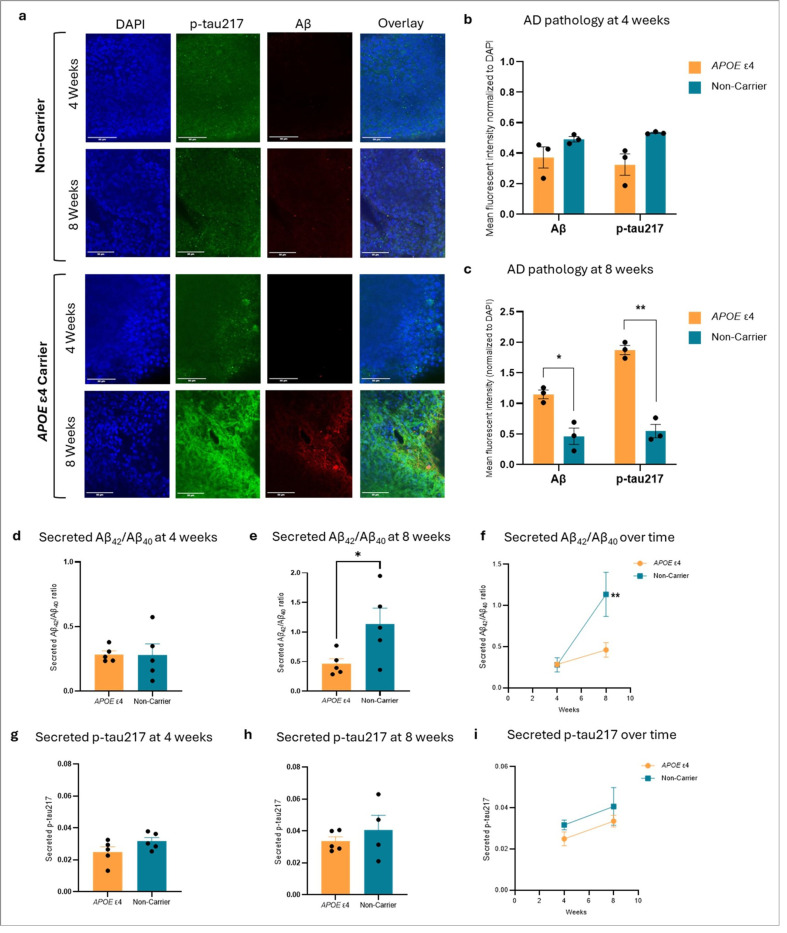
Alzheimer’s pathology development in induced pluripotent stem cell-derived cortical organoids of an *APOE* ε4 carrier and non-carrier. (**a**) Representative immunofluorescent confocal microscopy images of the presence of AD pathological markers p-tau217 and amyloid-β in cortical organoids from an *APOE* ε4 carrier and non-carrier at four and eight weeks of maturation. (**b**) Quantification of the mean fluorescent intensity of p-tau217 and amyloid-β, normalized to DAPI, in cortical organoids from an *APOE* ε4 carrier and non-carrier at four weeks of maturation (*n* = 3 technical replicates/group). (**c**) Quantification of the mean fluorescent intensity of p-tau217 and amyloid-β, normalized to DAPI, in cortical organoids from an *APOE*ε4 carrier and non-carrier at four weeks of maturation (*p = 0.0203, unpaired two-tailed t-test; **p = 0.0010, unpaired two-tailed t-test; *n*= 3 technical replicates/group). (**d,e,f**) ELISA analysis of secreted amyloid-β42/40 into the culture media from *APOE* ε4 carrier and non-carrier cortical organoids at (**d**) 4 weeks and (**e**) 8 weeks of maturation. There was no difference at 4 weeks between the *APOE*ε4 carrier and non-carrier whereas the non-carrier cortical organoids showed significantly (*p = 0.0432, unpaired t-test, n = 5 technical replicates/group) higher levels of secreted amyloid-β42/40 at 8 weeks. (**f**) The non-carrier cortical organoids showed significantly different levels of secreted amyloid-β42/40 over time relative to the *APOE* ε4 cortical organoids (** *p*= 0.0166, two-way ANOVA, n = 5 technical replicates/group). (**g,h,i**) ELISA analysis of secreted p-tau217 into the culture media from *APOE* ε4 carrier and non-carrier cortical organoids at (**g**) 4 weeks and (**h**) 8 weeks of maturation (*n* = 5 technical replicates/group). There were no significant differences between groups. (**i**) Time course of *APOE*ε4 carrier and non-carrier cortical organoid-secreted p-tau217 showing no significant changes over time (*n* = 5 technical replicates/group).

To address this, we generated cortical organoids ^15,16^ from induced pluripotent stem cell (iPSC) lines of two donors from the University of Kansas Alzheimer’s Disease Research Center cohort: a heterozygous *APOE* ε4 carrier who developed AD and a non-carrier control. To better model *APOE* ε4 immune interactions, iPSC-derived microglia from the same donors were integrated into cortical organoids (Methods; Extended Data Fig. 2). At four weeks maturation, neither *APOE* ε4 nor non-carrier organoids showed evidence of p-tau217 or amyloid-β accumulation (Fig. 3a,b) and no differences in secreted amyloid-β42/40 or p-tau217 (Fig. 3d,f,g,i). By eight weeks, however, *APOE* ε4 organoids exhibited marked accumulation of p-tau217 (t(2.5) = 4.48, *p* = 0.0203, unpaired two-tailed t-test; Fig. 3a,c) and amyloid-β (t(2.5) = 10.02, *p* = 0.0010, unpaired two-tailed t-test; Fig. 3a,c). This was accompanied by significantly reduced levels of secreted amyloid-β42/40 compared to non-carriers (t(8) = 2.40, p = 0.043; unpaired two-tailed t-test; Fig. 3e), consistent with amyloid-β retention in *APOE* ε4 carrier organoids. A significant time × *APOE* genotype interaction in amyloid-β42/40 secretion was also observed (F(1,8) = 9.12, p = 0.017; two-way ANOVA; Fig. 3f). Despite intracellular p-tau217 accumulation in *APOE* ε4 organoids at eight weeks, no significant differences were observed in secreted p-tau217 across groups or over time (Fig. 3g-i).

**Figure 4:**
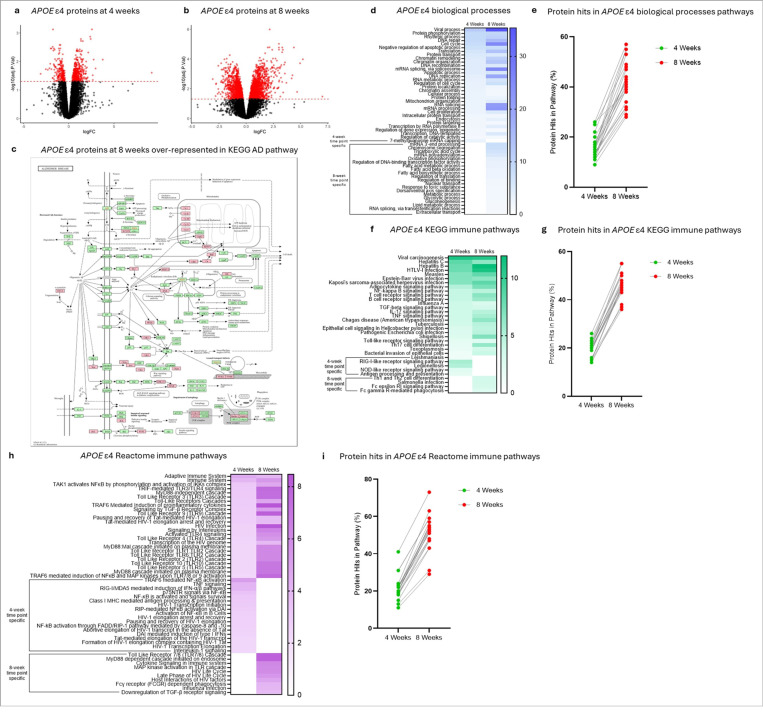
Characterization of the *APOE* ε4 proteome over time in stem cell-derived cortical organoids from an *APOE* ε4 carrier and non-carrier. (**a**) Volcano plot representing 642 significantly differentially expressed proteins (in red) in *APOE* ε4 cortical organoids at four weeks of maturation before the onset of AD pathologies; *n* = 4 technical replicates/group (see Supplementary Table 8 for a full list of proteins, adjusted *p*-values, and fold changes). (**b**) Volcano plot representing 2,655 significantly differentially expressed proteins (in red) in *APOE* ε4 cortical organoids at eight weeks of maturation after the onset of AD pathologies; *n* = 4 technical replicates/group (see Supplementary Table 8 for a full list of proteins, adjusted *p*-values, and fold changes). (**c**) KEGG pathway enrichment analysis of differentially expressed proteins in *APOE* ε4 cortical organoids at eight weeks of maturation showing significant (FDR = 0.00000211) enrichment in the AD pathway. Pink colored proteins represent those identified as differentially expressed proteins at eight weeks. (**d**) Significantly (FDR < 0.05) enriched PANTHER biological processes for *APOE*ε4 cortical organoids at four and eight weeks of maturation. The top enriched process across both timepoints was viral processes (FDR = 1.29E-12 and 3.99E-36, respectively). The scale bar represents −1og10(FDR). (**e**) Graph showing the change in the number of identified (“hit”) proteins in overlapping *APOE*ε4 PANTHER biological processes across cortical organoids at four and eight weeks of maturation. The y-axis represents the “hit” proteins expressed as a percentage of expected proteins (“hit” proteins in pathway / total number of proteins in pathway). (**f**) Significantly (FDR < 0.05) enriched KEGG immune pathways for *APOE* ε4 cortical organoids at four and eight weeks of maturation. Enriched pathways were reflective of processes involved in pro-inflammatory, cytokine, infection, and immune function and time point-specific pathways indicated a changing role of microglia from surveillance and immune sensing to activated and phagocytic. (**g**) Graph showing the change in the number of identified (“hit”) proteins in overlapping *APOE*ε4 KEGG immune pathways across cortical organoids at four and eight weeks of maturation. The y-axis represents the “hit” proteins expressed as a percentage of expected proteins (“hit” proteins in pathway / total number of proteins in pathway). (**h**) Significantly (FDR < 0.05) enriched Reactome immune pathways for *APOE* ε4 cortical organoids at four and eight weeks of maturation. Enriched pathways mirrored those seen in the KEGG analysis and further indicated a change in microglia over time from innate immune sensing and inflammatory priming to phagocytic, disease-associated microglia (DAM). (**i**) Graph showing the change in the number of identified (“hit”) proteins in overlapping *APOE* ε4 Reactome immune pathways across cortical organoids at four and eight weeks of maturation. The y-axis represents the “hit” proteins expressed as a percentage of expected proteins (“hit” proteins in pathway / total number of proteins in pathway).

**Figure 5:**
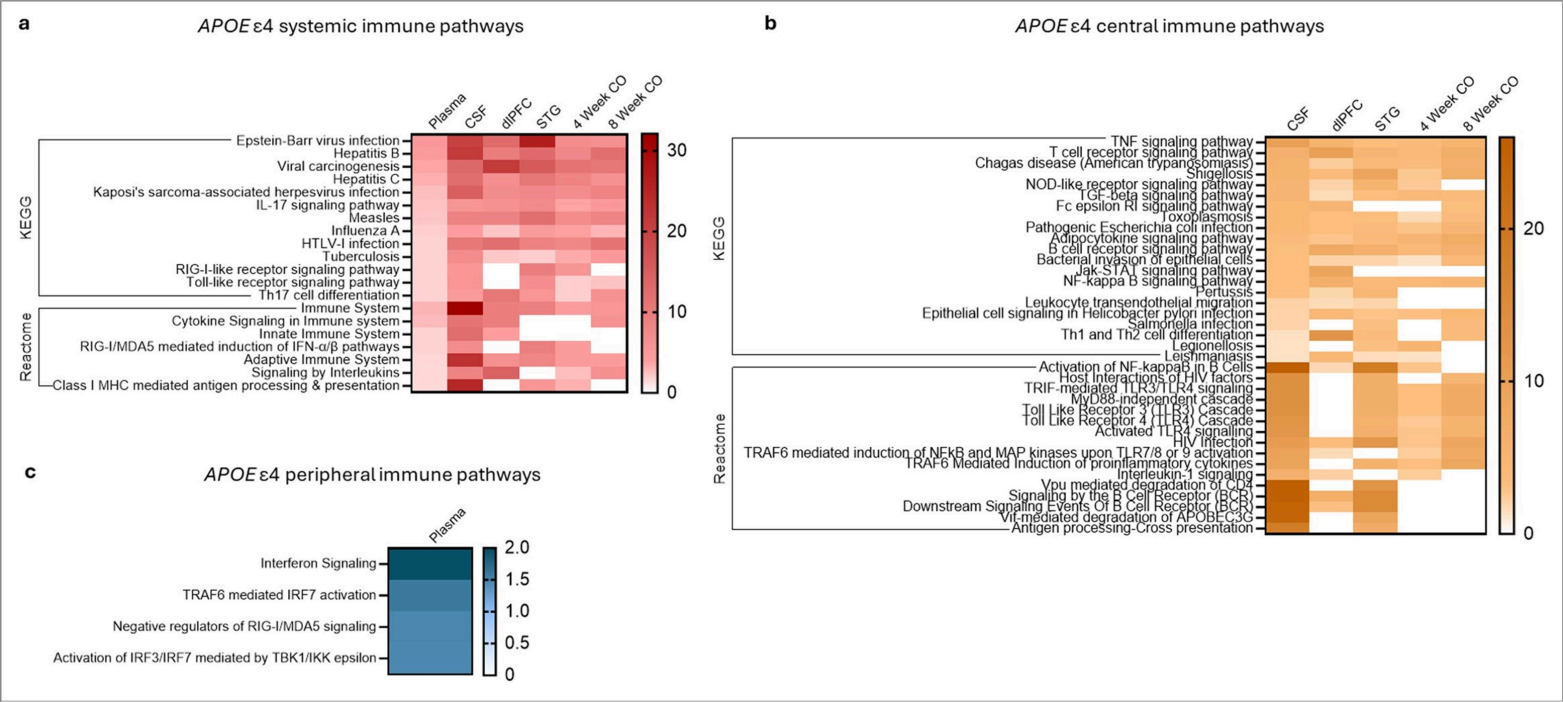
Cross-tissue comparison of immune pathways in *APOE* ε4 carriers. (**a**) Systemic *APOE*ε4 immune KEGG and Reactome pathways identified across the plasma, cerebrospinal fluid (CSF), dorsolateral prefrontal cortex (dlPFC), superior temporal gyrus (STG), and cortical organoids (CO). (**b**) Central nervous system-specific *APOE*ε4 immune KEGG and Reactome pathways identified across the CSF, dlPFC, STG, and CO. (**c**) Peripheral system-specific *APOE* ε4 immune KEGG and Reactome pathways identified only in the plasma. Scale bars represent −1og10(FDR). Abbreviations: AD: Alzheimer’s disease; CO: cortical organoids; CSF: cerebrospinal fluid; dlPFC: dorsolateral prefrontal cortex; STG: superior temporal gyrus.

At four weeks of maturation, *APOE* ε4 cortical organoids lacked hallmark AD pathology, providing an opportunity to study early pre-pathological processes. To investigate how the *APOE* ε4 proteome evolves with disease onset, we performed label-free mass spectrometry on 10,828 proteins from *APOE* ε4 and non-carrier organoids at four and eight weeks. At four weeks, 642 proteins were differentially expressed between *APOE* ε4 and non-carriers, increasing to 2,655 by eight weeks (adjusted p < 0.05; Supplementary Table 8; Fig. 4a-b). KEGG enrichment confirmed absence of AD pathway proteins at four weeks, with significant enrichment emerging by eight weeks (FDR = 2.1 × 10^−6^ Fig. 5c). Consistent with our plasma, CSF, and brain findings, viral processes were the most enriched biological process at both time points (PANTHER; Fig. 4d; Supplementary Table 9). Pathways evident at four weeks were largely exacerbated at eight weeks, as reflected by increased protein representation (Fig. 4e). New eight-week-specific enrichments included pathways related to RNA metabolism, oxidative phosphorylation, lipid metabolism, glycolysis, and stress responses, potentially reflecting changes secondary to accumulating pathology (Fig. 4d; Supplementary Table 9).

KEGG immune pathway analysis showed substantial overlap between early and late time points, with progressive intensification over time (Fig. 4f-g; Supplementary Table 9). Early pathways reflected innate immune sensing and antigen presentation, suggesting early APOE ε4 microglial priming. By eight weeks, pathways were reflective of chronic innate activation, phagocytosis, and adaptive immune responses, consistent with a transition to a disease-associated microglial (DAM) phenotype in response to pathology (Fig. 4f; Supplementary Table 9). Reactome enrichment further supported this finding. Early time points were dominated by NF-κB, type I interferon, and MHC class I antigen presentation while late time points showed increased pathways for phagocytosis, cytokine signaling, and potential astrocyte involvement via TGF-β signaling (Fig. 4h; Supplementary Table 9). Pathways common to both time points showed progressive activation over time, as evidenced by increased numbers of contributing proteins (Fig. 4i).

Collectively, these data demonstrate that *APOE* ε4 cortical organoids display an early pro-inflammatory, viral-related immune phenotype prior to the onset of AD pathology, which progressively intensifies with maturation and disease development. The observed transition from innate immune priming to sustained activation and phagocytic responses is consistent with the emergence of DAM. These results highlight cortical organoids as a valuable model for dissecting temporal immune mechanisms underlying *APOE* ε4-driven AD pathogenesis.

### Cross-tissue proteomic analysis reveals systemic as well as central- and peripheral-specific dysregulated pathways in *APOE* ε4 carriers

Thus far, we independently characterized the proteomes of plasma, CSF, two brain regions, and cortical organoids from *APOE* ε4 carriers and non-carriers. While broad enrichment of pro-inflammatory immune pathways was observed across compartments, it remained unclear which *APOE* ε4-associated molecular changes were systemic versus compartment-specific. To address this, we performed a cross-tissue comparison of enriched KEGG and Reactome immune pathways to delineate molecular processes that are systemic, CNS-specific, or peripheral in nature.

Systemic *APOE* ε4 pathways were broadly representative of conserved innate and adaptive immune responses to diverse viral and bacterial infections and pro-inflammatory stimuli, as well as generalized cytokine signaling and antigen presentation (Fig. 5a). Enrichment was also observed for pattern recognition receptor signaling, interferon responses, and MHC class I antigen presentation, core elements of viral sensing, antiviral immunity, and systemic immune activation (Fig. 5a). In contrast, CNS-specific pathways were associated with more complex immune functions, including adaptive immune signaling through T and B cells, intracellular pathogen responses, chronic inflammatory responses via TNF, NF-κB, and JAK-STAT pathways, and immune modulation (Fig. 5b). Enrichment of pathways involved in immune-microbe interactions at tissue barriers and immune cell recruitment was also observed, potentially reflecting infiltrating adaptive immune cells or blood-brain barrier (BBB)-associated immune processes (Fig. 5b). Notably, five central *APOE* ε4-enriched pathways were absent in cortical organoids at both time points (Fig. 5b), suggesting that these pathways, particularly those requiring mature T/B cell interactions, antigen presentation, and immune cell crosstalk, cannot be fully recapitulated in organoids due to the absence of adaptive, peripherally derived immune cells. Only four peripheral-only pathways were identified in plasma, primarily involving the ne-tuning and regulation of interferon-driven transcriptional responses (Fig. 5c).

These results demonstrate that the *APOE* ε4 pro-inflammatory immune phenotype includes both systemic and CNS-specific components, with core antiviral and innate immune pathways shared across tissues and more complex adaptive immune and BBB-associated processes enriched in the CNS. The absence of certain adaptive pathways in organoids further underscores the importance of immune cell interactions in shaping the *APOE* ε4 CNS immune environment. Together, these findings highlight a multi-tissue, pro-inflammatory immune phenotype that may contribute to *APOE* ε4-driven AD risk.

### Short-term treatment with an anti-inflammatory ketogenic diet can modulate the pro-inflammatory phenotype of *APOE* ε4 carriers

*APOE* ε4 carriers have a pro-inflammatory phenotype that spans both the periphery and CNS. To test whether this phenotype is modifiable, we analyzed plasma samples from a 12-week trial of a medical ketogenic diet in 15 individuals with mild AD (*n* = 9 *APOE* ε4 carriers, n = 6 non-carriers; Supplementary Table 10). Medical ketogenic diets, focused on increasingly quality fat intake and reducing carbohydrate consumption to promote production of ketone bodies are widely regarded as a gold-standard systemic anti-inflammatory diet ^17^. They have demonstrated treatment benefits for epilepsy, general neurological conditions including AD, and cancers ^18–21^. The intervention increased serum β-hydroxybutyrate in both groups, confirming ketone body induction (Supplementary Table 10).

**Figure 6:**
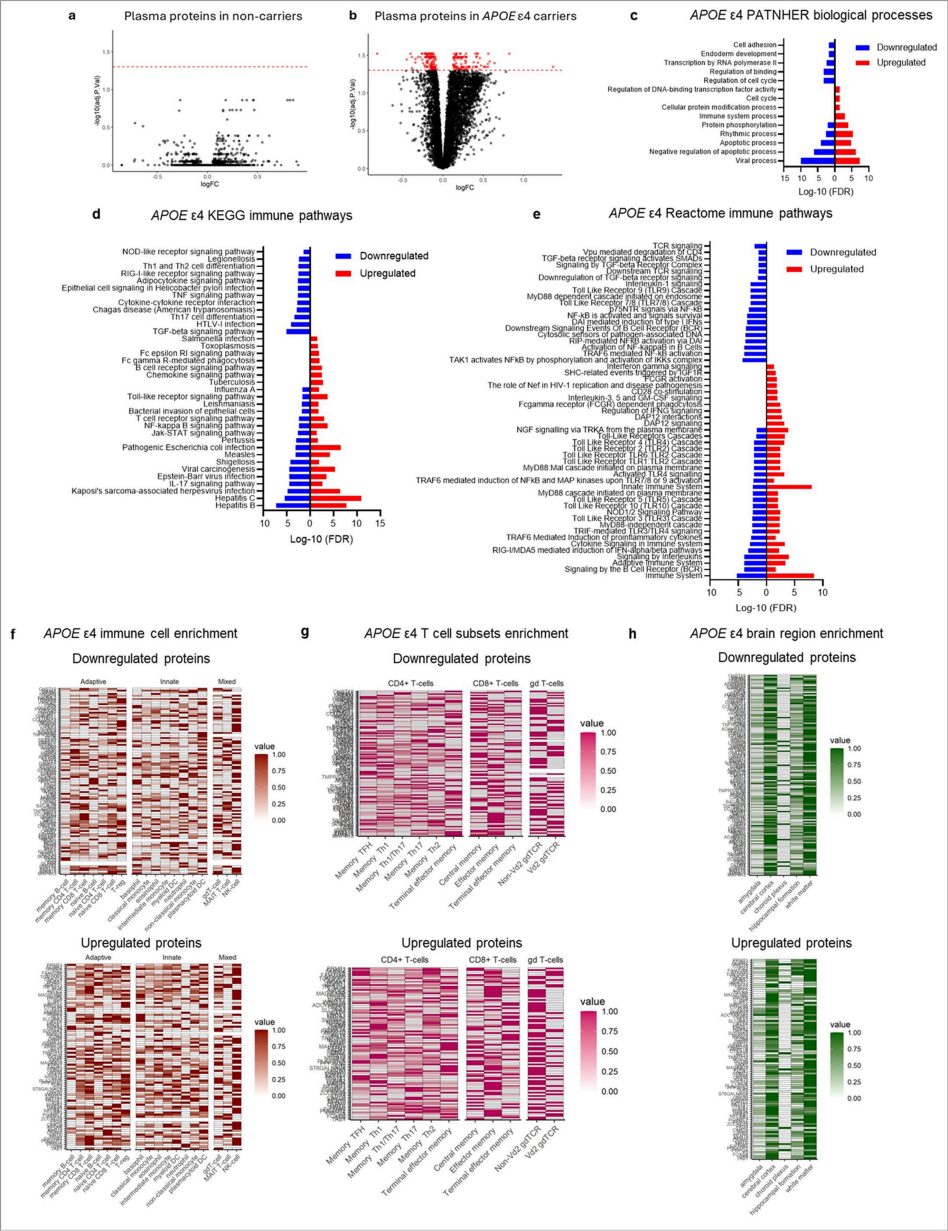
Characterization of the plasma proteome of Alzheimer’s patients after 12 weeks of an anti-inflammatory medical ketogenic diet from the Therapeutic Diets in Alzheimer’s Disease clinical trial. (**a**) Volcano plot representing no significantly differentially expressed proteins in non-carriers at the end of the 12-week study; *n* = 9 *APOE* ε4 carriers and *n* = 6 non-carriers. (**b**) Volcano plot representing 264 significantly (adjusted *p*-value < 0.05) differentially expressed proteins in *APOE* ε4 carriers at the end of the 12-week study; *n* = 9 *APOE* ε4 carriers and *n* = 6 non-carriers (see Supplementary Table 11 for a full list of proteins, adjusted *p*-values, and fold changes). (**c**) PANTHER biological processes analysis of pathways enriched for up- or down-regulated differentially expressed proteins in *APOE* ε4 carriers. (**d**) KEGG immune pathway analysis of pathways enriched for up- or down-regulated differentially expressed proteins in *APOE* ε4 carriers. (**e**) Reactome immune pathway analysis of pathways enriched for up- or down-regulated differentially expressed proteins in *APOE* ε4 carriers. (**f**) Enrichment of significantly up- or down-regulated *APOE* ε4 proteins across distinct immune cell subsets. Downregulated proteins were primarily enriched in T-regs and NK cells. Upregulated proteins were primarily enriched across memory CD8^+^ T cells, naïve CD8^+^ T cells, T-regs, and NK cells. There was also more enrichment across the spectrum of innate immune cells relative to *APOE* ε4 downregulated proteins. (**g**) Given that we found high enrichment for T cells, broadly, we did a further enrichment for distinct T-cell subsets. Downregulated *APOE* ε4 proteins were primarily enriched in effector memory and central memory CD8^+^ T cells and non-Vδ2 γδ T cells. Upregulated *APOE* ε4 proteins were similarly enriched for effector memory CD8^+^ T cells and non-Vδ2 γδ T cells as well as CD4^+^ T cells including memory TFH and terminal effector memory. (**h**) Enrichment of significantly up- and down-regulated *APOE* ε4 proteins across commonly affected brain regions in AD, with the highest enrichment for white matter. Enrichments are based on single cell RNA-sequencing data from the Human Protein Atlas ^12–14^ and plots show mix-max scaling of protein-coding transcripts per million for each identified *APOE* ε4 plasma protein. Abbreviations: AD: Alzheimer’s disease; DC: dendritic cell; TFH: T follicular helper T cells.

Plasma proteomic profiling (7,595 proteins via SomaScan 7k) across baseline and study endpoint revealed no significant changes in non-carriers (Fig. 6a). In contrast, *APOE* ε4 carriers had 264 differentially expressed proteins (161 downregulated, 103 upregulated; adjusted *p* < 0.05; Fig. 6b; Supplementary Table 11). PANTHER analysis identified viral processes as the top enriched biological process across both up- and downregulated proteins (Fig. 6c; Supplementary Table 12). A KEGG immune pathway analysis revealed that the medical ketogenic diet led to immune remodelling in *APOE* ε4 carriers rather than broad suppression or activation. Pathways enriched for both up- and downregulated proteins included those involved in innate and adaptive pathways (Fig. 6d). Pathways enriched in upregulated proteins were associated with adaptive immunity, phagocytosis, and chemotaxis, suggesting enhanced immune surveillance. Downregulated proteins, however, mapped to chronic inflammation and metabolic-immune signaling, indicating reduced chronic pro-inflammatory signaling (Fig. 6d; Supplementary Table 12). These findings were mirrored by a Reactome pathway analysis. This showed a bidirectional regulation of TLR and cytokine signaling, with selective upregulation of interferon responses and Fc-mediated phagocytosis, and downregulation of NF-κB, TGF-β, TCR, and IL-1 signaling pathways (Fig. 6e; Supplementary Table 12). Cell type enrichment analysis showed that both up- and downregulated proteins were mapped to T-regs, NK cells, memory CD8^+^ T cells, and non-Vδ2 γδ T cells, suggesting functional reprogramming within these populations rather than uniform activation or suppression (Fig. 6f,g). Notably, both protein sets were also enriched in white matter, highlighting that the CNS may have similar immune shifts in response to the ketogenic diet.

Taken together, these findings indicate that a medical ketogenic diet leads to reprogramming rather than uniform activation or suppression of immune cell populations. Protein expression patterns and pathway analyses suggest a shift in T-regs and NK cells from pro-inflammatory or cytotoxic states toward more regulatory, tissue supportive phenotypes. Similarly, memory CD8^+^ T cells likely have reduced signatures of chronic activation alongside enhanced effector memory features, consistent with restored adaptive immune competence. Non-Vδ2 γδ T cells also displayed bidirectional modulation, suggesting altered roles at the innate-adaptive interface and reduced IL-17-associated inflammatory signaling.

## Discussion

Our study provides the most comprehensive cross-tissue characterization to date of the pro-inflammatory immune phenotype associated with *APOE* ε4, integrating large-scale clinical proteomic data with experimental validation in iPSC-derived cortical organoids and a dietary intervention trial. We demonstrate that *APOE* ε4 carriers exhibit a conserved, allele dose-dependent pro-inflammatory immune signature across plasma, CSF, and brain tissue, independent of AD status. Importantly, this immune phenotype was also recapitulated in cortical organoids, emerging prior to the development of amyloid-β or tau pathology. This indicates that key features of the *APOE* ε4 immune signature arise early in disease pathogenesis and reflect an intrinsic, genotype-driven mechanism of immune dysregulation. Cross-tissue analyses further revealed a core set of systemic immune pathways involving broad innate and adaptive responses to infection, alongside distinct tissue-specific signatures, including inflammatory signaling and BBB-related interactions within the CNS. Notably, we show that this immune phenotype is not fixed. Short-term treatment with a medical ketogenic diet in *APOE* ε4 carriers with AD partially reversed pro-inflammatory signatures, promoting more regulatory and tissue-supportive immune states. Together, these findings illuminate how peripheral and central tissue environments modulate *APOE* ε4-driven immune responses, provide new mechanistic insight into AD pathogenesis, and support the development of precision biomarkers and immunomodulatory interventions targeting early-stage disease processes.

Our results are consistent with and extend prior work. Multiple studies have reported heightened peripheral immune responses in *APOE* ε4 carriers, including increased cytokine production following TLR2/4/5 activation ^3^, elevated TNF-α and IL-6 levels after a lipopolysaccharide (LPS) challenge ^4^, increased cytokine levels ^6,22–24^, and enhanced susceptibility to systemic inflammatory stressors such as sepsis ^4^. Similarly, transcriptomic and epigenomic profiling of PBMCs has demonstrated broad dysregulation of innate immune pathways, altered NF-κB activation, increased chromatin accessibility in CD14^+^ monocytes, and clonally expanded CD8^+^ effector memory T cells ^25^. Our plasma *APOE* ε4 proteomic signature mirrors these findings, with robust enrichment for innate immune and antiviral responses, including TLR-NF-κB and interferon signaling cascades. Moreover, we observed allele dose-dependent effects, consistent with prior reports of exacerbated immune dysfunction in homozygous *APOE* ε4 carriers ^3,25^. Notably, our findings also align with the emerging hypothesis that *APOE* ε4 carriers exhibit increased susceptibility to viral reactivation and higher viral titers and that this plays a role in the development of AD ^26^. The strong enrichment of viral response pathways we find across plasma, CSF, and brain in our study supports this hypothesis. Prior studies measuring C-reactive protein (CRP) levels in *APOE* ε4 carriers have yielded mixed results, with some reporting decreased baseline CRP ^22,27–29^, while others report increased associated brain atrophy ^30^. Our findings suggest that *APOE* ε4-driven chronic immune dysregulation is not adequately captured by individual markers of acute inflammation such as CRP. A limitation of our study is the lack of longitudinal data. Although we show that the *APOE* ε4 immune phenotype is present in cognitively unimpaired individuals, future work should investigate whether this signature evolves with disease progression in *APOE* ε4 carriers.

In the CNS, our findings offer new mechanistic insight into how *APOE* ε4 may prime resident immune cells, particularly microglia, toward a disease-associated phenotype. Prior studies have demonstrated that *APOE* ε4 microglia exhibit impaired phagocytosis ^11^, disrupted autophagy with lipid droplet accumulation ^9,10^, and exaggerated inflammatory responses to amyloid-β ^10,11^. Our CSF and brain proteomic data con rm upregulation of inflammatory pathways (NF-κB, TNF signaling), consistent with a chronically activated or DAM-like state and reveal enrichment of BBB-related immune pathways. These findings align with evidence of *APOE* ε4-associated BBB dysfunction ^31,32^ and increased infiltration of peripheral immune cells such as IL-17^+^ neutrophils ^7^. Notably, we observed stronger pro-inflammatory enrichment in the STG compared to the dlPFC. This likely reflects the greater vascularization and CSF-blood interface in the STG, which may facilitate immune interactions.

A major strength of our study is the use of patient-derived cortical organoids to model *APOE* ε4-driven immune changes in human neural tissue before AD pathology emerges. While prior studies of *APOE* ε4 immunopathology have largely focused on peripheral immune cells ^3–7,22–25,30,33,34^, postmortem brain tissue ^8,35^, or single iPSC-derived cell types ^9–11^, our organoid model enables longitudinal study of *APOE* ε4 effects within a more complex network. We experimentally confirmed early activation of inflammatory pathways, including NF-κB and DAM-like signatures, prior to amyloid-β or tau pathology. This provides direct evidence that *APOE* ε4 immune dysregulation is intrinsic and genotype-driven, rather than a secondary response to existing AD pathology. Moreover, the fact that *APOE* ε4 immune activation precedes amyloid-β and tau accumulation supports the view that these hallmark pathologies may be downstream consequences rather than primary disease drivers in *APOE* ε4 carriers. These insights challenge current therapeutic paradigms focused solely on amyloid-β and tau, targets that, despite effective clearance, have shown limited clinical bene t and in some cases worsening disease ^36–39^. Instead, our findings suggest that tempering the persistent *APOE* ε4-driven immune response through immunomodulation or anti-inflammatory approaches will be critical for altering disease risk and progression in this high-risk population. Importantly, this aligns with emerging evidence that vaccinations reduce AD risk ^40,41^, including the shingles vaccines Zostavax ^42^ and Shingrix ^43^, potentially by reducing reactivation of varicella zoster virus and/or inducing a virus-specific immunomodulatory effect ^42^. Targeting immune dysregulation should therefore be prioritized in future precision medicine strategies for AD.

Our finding that a medical ketogenic diet partially reversed the *APOE* ε4 immune phenotype has important implications for therapeutic development. Twelve weeks of treatment led to the downregulation of chronic inflammatory signaling pathways, including NF-κB, IL-1, and TGF-β and upregulation of regulatory and phagocytic functions across T-regs, NK cells, and memory CD8^+^ T cells. These changes suggest a shift away from maladaptive, chronic inflammation toward a more balanced and functional immune state. This supports the idea that immune modulation may be a viable disease-modifying strategy, particularly if implemented early, before irreversible pathology has accumulated. Future studies would bene t from larger-scale clinical trials of a medical ketogenic diet as well as other immunomodulatory therapies before the onset of AD in *APOE* ε4 carriers.

One limitation of our study is that cross-platform differences limited direct comparisons of APOE ε4 tissue proteomes. Plasma and CSF were profiled using SomaScan aptamer-based technology, while TMT and label-free mass spectrometry were used for brain and organoid analyses, respectively. As a result, we focused on pathway-level rather than protein-level comparisons across samples. However, this limitation is also a strength. The consistency of pathway-level findings across multiple platforms provides orthogonal validation of the robustness and generalizability of our results. Moreover, our study demonstrates that aptamer-based approaches, despite known limitations in proteoform sensitivity ^44^, can reliably capture *APOE* ε4-related pathway alterations. Future work using harmonized proteomic platforms will further refine these insights.

In conclusion, our findings establish that *APOE* ε4 drives a conserved pro-inflammatory immune phenotype that emerges early, prior to the development of hallmark AD pathology. These results advance the mechanistic understanding of *APOE* ε4-mediated AD risk and challenge the prevailing amyloid-β- and tau-centric therapeutic approaches, especially for *APOE* ε4 carriers. By demonstrating that immune dysregulation is an intrinsic, upstream feature of *APOE* ε4, and showing that it is modifiable, our study underscores the need to prioritize immunomodulatory and anti-inflammatory strategies in precision medicine approaches for this high-risk group. Moving forward, longitudinal studies and harmonized proteomic platforms will be critical to further delineate the temporal evolution of the *APOE* ε4 immune signature and to guide the development of targeted interventions aimed at altering disease trajectories in *APOE* ε4 carriers.

## Methods

### Participants

#### Global Neurodegeneration Proteomics Consortium (GNPC) cohort.

The GNPC cohort is the largest collection of proteomic data for individuals with neurodegenerative diseases and non-impaired controls from >20 clinical sites from across the USA, UK, and Europe. In the current study, we included non-impaired controls (n = 6,672) and individuals with AD (n = 3,455). Across both groups, n = 6,107 were non-*APOE* ε4 carriers and n = 4,767 had at least one *APOE* ε4 allele (Supplementary Table 1). AD was clinically diagnosed within each study site as previously described including Clinical Dementia Rating (CDR) score of ≥ 1, Mini-Mental State Exam (MMSE) ≤ 24, and/or Montreal Cognitive Assessment (MOCA) score of ≤ 23 ^45^. Individual plasma and CSF samples, demographic, and clinical variables were collected at a single timepoint (Supplementary Table 1). Participants from each study site in the GNPC cohort provided written informed consent and studies were approved by the relevant institution’s ethics committee ^45^.

#### Accelerating Medicines in AD Partnership (AMP-AD) Diverse Cohorts study.

The AMP-AD Diverse Cohorts study is a cross-consortium project that created harmonized high-throughput multi-omic data for post-mortem brain tissue samples. AD cases were pathologically defined as described elsewhere ^46^. Samples from the dorsolateral prefrontal cortex (dlPFC) and superior temporal gyrus (STG) were collected across four institutions: Mayo Clinic, Rush University, Mount Sinai University Hospital, and Emory University. In the present study, we included dlPFC data from *n* = 190 non-impaired controls and *n* = 530 AD cases. For the STG, *n* = 49 non-impaired controls and *n* = 161 AD cases were included. Supplementary Table 6 lists the demographic characteristics for the included donors. Participants from each study site in the AMP-AD Diverse Cohorts study provided written informed consent and studies were approved by the relevant institution’s ethics committee.

#### University of Kansas Alzheimer’s Disease Research Center donors for cortical organoids.

Two donors from the University of Kansas Alzheimer’s Disease Research Center provided post-mortem skin samples. The non-carrier donor was a 72-year-old female with an *APOE* ε3/ε2 genotype and *APOE* ε4 carrier donor was an 84-year-old female with an *APOE* ε3/ε4 genotype.

#### Therapeutic Diets in Alzheimer’s Disease (TDAD) clinical trial.

The TDAD clinical trial is a single-blind, randomized, controlled study of the effects of a 12-week medical ketogenic dietary intervention in patients with mild AD. Participants were recruited through the Clinical Translational Science Unit at the University of Kansas Medical Center Clinical Research Center. Fifteen participants (*n* = 9 *APOE* ε4 carriers and *n* = 6 non-carriers) were given a ketogenic diet intervention. All participants met the criteria for probable AD based on the National Institutes of Aging-Alzheimer’s Association (NIA-AA) criteria ^47^ and had a baseline CDR of 0.5 to 1 (see Supplementary Table 10 for demographics). Participants with moderate to severe AD, including those residing in a nursing home or dementia special care unit, were excluded from the trial based on our previous experience showing that these individuals have a low retention rate to dietary interventions ^21^. Participants with serious medical conditions, those participating in another clinical trial, women of child-bearing capacity who seek to become pregnant, and non-English speakers were also excluded. Table 1 lists the inclusion and exclusion criteria for the TDAD clinical trial.

Ketogenic diets focus on increasing quality fat intake and reducing carbohydrate consumption to promote the production of ketone bodies, mimicking the effects of prolonged fasting and vigorous exercise ^17^. Macronutrients were maintained at approximately 70% fat, 20% protein, and ≤ 10% carbohydrates. A 1:1 emulsified medium chain triglyceride (MCT) oil was also provided to participants in the ketogenic diet group to increase fat intake, enhance ketosis, and increase palatability in line with other medical ketogenic diets ^21^. To reduce gastrointestinal side effects from the MCT, the supplement dose increased on a weekly basis across a four-week period, starting from an intake level corresponding to 10% of total fat energy and gradually increasing to 40%. The Mifflin-St. Jeor equation was used to calculate target energy goals ^48^. All participants received weekly dietary counselling from the study dietitian. Participants also received dietitian-created ketogenic diet educational material and a manual. Manuals included sample menus, recipes, and strategies for maintaining their respective diet during travel or holidays.

Participants’ APOE genotype, medical history, medication use, comorbidities, smoking, and alcohol intake were recorded at their initial screening visit. Blood samples were taken at two time points, a pre-intervention baseline and post-intervention (12 weeks), for plasma proteomics and to measure depth of ketosis using a routine serum β-hydroxybutyrate assay performed commercially by Quest Diagnostics (Lenexa, KS). Participant demographic characteristics are listed in Supplementary Table 10.

The TDAD clinical trial was approved by the University of Kansas Center IRB (STUDY00143457). Prior to participating in the study, all participants provided informed consent. The trial was registered on ClinicalTrials.gov under registration number NCT03860792.

### Cortical Organoids

#### Induced pluripotent stem cell derivation.

Induced pluripotent stem cells (iPSCs) were generated from broblast samples from both donors using a CytoTune-iPSC 2.0 Sendai Reprogramming kit (ThermoFisher Scientific, A16517) according to the manufacturer’s instructions. iPSCs were plated down into 6-well plates pre-treated with 0.08mg/ml Matrigel (Corning, 356234) in Dulbecco’s Phosphate-Buffered Saline (DPBS). StemFlex medium (Gibco, A3349401) with 100U/ml penicillin-streptomycin added (Gibco, 15140122) was added to each well and changed every two days. iPSCs were passaged once a week using ReLeSR (StemCell Technologies, 15140122) as per the manufacturer’s protocol. Following each passage, medium was supplemented with 0.01mM Y-27632 (StemCell Technologies, 72302) for 24 hours.

#### iPSC-derived microglia differentiation.

iPSCs were differentiated into hematopoietic progenitor cells (HPCs) using a STEMdiff Hematopoietic kit (StemCell Technologies, 5310) as per the manufacturer’s instructions. First, iPSCs were passaged into HPC Medium A in 24-well plates pre-treated with 0.04mg/ml Matrigel. After three days, they were cultured with HPC Medium B for nine more days. Mature HPCs were then collected and passaged into STEMDiff Microglia Differentiation medium (StemCell Technologies, 100–0019) as per the manufacturer’s instructions. Fresh media was added every other day for 24 days and cells were passaged once on day 12. After differentiation, microglia were matured in BrainPhys medium with N2-A and SM1 supplements (StemCell Technologies, 05793) for four days prior to integration into embryoid bodies.

#### Cortical organoid development.

iPSCs were first passaged three times to stabilize the line. Embryoid bodies were generated in ultra-low attachment dishes (Corning, 3261) using StemFlex medium supplemented with 100U/ml penicillin-streptomycin and 0.01mM y-27632 ROCK inhibitor. Media was changed every two days. Embryoid body medium was replaced into N2/B27 medium (Methods Table 2) and transferred into a 24-well plate. Mature microglia were added to each well at a concentration of 0.8 × 10^5^ cells per well. Fresh N2/B27 media was added every two days and organoids were passaged once every two weeks. Cortical organoids were aged for either four or eight weeks.

#### Immunofluorescent staining.

At each timepoint, cortical organoids were removed from the media and washed in DPBS three times for 10 minutes. They were then fixed in 4% paraformaldehyde (Sigma-Aldrich, 158127) for 20 minutes, washed in DPBS once for 10 minutes, and stored in cyroprotectant (30% glycerol (Sigma-Aldrich, G5516), 30% ethylene glycol (Sigma-Aldrich, 324558), 40% PBS) at −20°C. Organoids were washed in PBS three times for 10 minutes and incubated with blocking solution (5% donkey serum (Sigma-Aldrich, D9663) or 5% bovine serum albumin (BSA), 0.1% Triton X-100 (Sigma-Aldrich, X100), and PBS) overnight at 4°C. Organoids were then incubated with primary antibodies (Methods Table 3) made up in blocking solution for two days at 4°C. They were then washed three times with PBS for 10 minutes followed by an overnight incubation with secondary antibodies (Methods Table 3) made up in blocking solution at 4°C. This process was repeated for each primary antibody. The MAP2, GFAP, and IBA1 stained organoids (Extended Data Fig. 2) were co-stained for DAPI using NucBlue Live ReadyProbes (Invitrogen, R37605) as per the manufacturer’s instructions and incubated for three days at 4°C. The amyloid-β and p-tau217 organoids (Fig. 3) were co-stained for DAPI using ProLong Gold AntiFade with DAPI (Invitrogen, P36931).

Prior to imaging, stained cortical organoids were washed three times in PBS for 10 minutes and incubated with CytoVista 3D Cell Culture clearing reagent (Invitrogen, V11315) overnight at 4°C. To visualize and quantify amyloid-β and p-tau217 staining, whole cortical organoids were imaged using a Nikon TI2-E inverted microscope attached to a Yokagawa CSU-W1 spinning disk confocal with SoRa super-resolution. Images were acquired with a Hamamatsu Fusion BT camera using Nikon NIS-Elements Advanced Research software. To visualize and quantify MAP2, IBA1, and GFAP staining, whole cortical organoids were imaged using a Marianas 3i Spinning Disk Confocal microscope with Super-Resolution by Optical Re-Assignment (SoRa). Representative images were taken using Fiji ImageJ v1.54p ^49^.

#### ELISA for secreted Aβ_42_/Aβ_40_ and p-tau217.

Media was collected from cortical organoids at four and eight weeks of age and proteins were extracted using acetone precipitation. Briefly, 1ml of ice-cold acetone (cooled to −20°C) was added to 0.5ml of collected media in a tube. Tubes were vortexed, incubated for 60 minutes at −20°C, and then centrifuged for 10 minutes at 15,000 × g. The supernatant was removed and pellets were washed in 70% ethanol before being resuspended in 8M urea. Secreted human amyloid-β_40_ and amyloid-β_42_ were measured with an ELISA as per the manufacturer’s instructions (ThermoFisher, KHB3481 and KHB3544). Secreted human p-tau217 was also measured with an ELISA as per the manufacturer’s instructions (Cell Signaling Technology, 59672). All samples were diluted 1:5 and concentrations were normalized to protein content measured via Pierce BCA protein assay (ThermoFisher, 23225). Graphs were made in GraphPad Prism v10.5.0.

### Proteomics

#### GNPC cohort SomaScan assay.

Proteomics of plasma and CSF samples from the GNPC cohort was done using the SomaScan v4.1 assay (SomaLogic). The SomaScan assay detects approximately 7,000 proteins using aptamer-based technology called slow off-rate modified aptamers (SOMAmers). These contain chemically modified nucelotides that bind with high specificity and affinity to target proteins ^50,51^. Raw data is provided by SomaLogic following standardization, normalization, and calibration, including adaptive normalization by maximum likelihood (ANML). Protein measurements are provided in relative fluorescent units (RFU). Prior to their inclusion in the GNPC dataset, aptamers are mapped to Uniprot. Details on the creation and harmonization of the GNPC dataset are described elsewhere ^45^. Data from the GNPC dataset was log2 transformed and standardized prior to analyses in the current study and was done separately on training and testing datasets.

#### AMP-AD Diverse Cohorts study TMT quantitation.

Proteomics on post-mortem dlPFC and STG homogenates was done using TMT mass spectrometry as previously described ^46^. Using a conservative approach, we only included proteins with ≤30% missing values across samples and imputed these values using the median. Batch effects were accounted for by fitting a linear regression model, as in the original study ^46^.

#### Cortical organoids label free mass spectrometry.

At 4 and 8 weeks of age, cortical organoids were snap frozen in liquid nitrogen and stored at −20 °C. To prepare samples for mass spectrometry, 0.2% n-dodecyl-β-D-maltoside (DDM) in 50 mM triethylammonium bicarbonate (TEAB) with 5 mM Tris(2-carboxyethyl)phosphine (TCEP) was added to each organoid. A pestle suitable for a 0.5 mL tube attached to a drill was used to homogenize the organoids for 10 s. The tubes were incubated at 85 °C for 10 min, then cooled in incubated with 10 mM iodoacetamide for 30 min at 22 °C. The samples were frozen in dry ice and then lyophilized. To each sample was added 10 μL of 0.1% DDM in 50 mM TEAB with 2.5 mM TCEP, 0.5 ug of LysC (WAKO Fuji lm) and 1 ug of trypsin (Sigma, trypzean). The samples were incubated at 42 °C for 2 h, then a further 0.5 ug of trypsin was added and incubated at 33 °C for 4 h. Each sample was acidi ed with 0.2 μL of formic acid and diluted with 30 μL of 0.1% tri uoroacetic acid and then desalted using the STAGEtip method ^52^.

Each sample was analyzed by LC-MS/MS using the Vanquish Neo system and Astral Orbitrap mass spectrometer (ThermoFisher Scientific). Samples were loaded onto a Pepmap Neo C18 5 μm particle 5 mm long by 300 μm inside diameter trap column (ThermoFisher Scientific) at up to 10 μL/min and at a maximum pressure of 800 bar. They were then eluted through a 15 cm long 150 μm inside diameter Pepmap C18 EASY-spray column with 2 μm 100 Å particles (ThermoFisher Scientific). The column was heated to 40 °C using the EASY-spray ion source operating a 1.9 kV. The S lens radio frequency level was 40 and capillary temperature was 280 °C.

The liquid chromatography used buffer A (solution of 0.1% formic acid) and buffer B (0.1% formic acid and 99.9% acetonitrile). After loading the sample in 3.6% buffer A, the gradient at 1 μL/min was from 7.2% to 25.2% buffer B in 19.7 min, to 31.5% buffer B in 3.7 min, to 49.5% buffer B in 0.4 min and to 90% buffer B at 3 μL/min in 0.5 min and held for 0.7 min. MS acquisition was for 26 min. The MS scans in the orbitrap analyzer were at a resolution of 240,000 with automatic gain control set to 5,000,000 and a maximum ion time of 3 ms for m/z 380 to 980. The data-independent acquisition MS/MS scans with a window of 2 m/z in the Astral analyser had automatic gain control at 50,000 for a maximum ion time of 3 ms. The loop was controlled to 0.6 seconds. The MS/MS scan range was 150–2000 m/z. The normalized collision energy was 25.

The raw LC-MS/MS data was processed with DIA-NN v1.9.2 ^53^. The *Homo Sapiens* reference proteome downloaded Feb 24 2025 with 20,644 genes, using canonical sequences only, was used to create an *in silico* library for peptide-spectrum matching. N-terminal methionine excision was allowed. Carbamidomethyl (C) was a fixed modification. Peptide length was 7–30. Initial mass accuracy was 10 ppm and MS1 accuracy was 4 pm. Digestion was set to trypsin/P with a maximum of 1 missed cleavage. Precursor false discovery rate was 1%. Match between runs was disabled. Heuristic protein inference was enabled, as were all other default algorithm settings for DIA-NN v1.9.2.

#### TDAD clinical trial SomaScan assay.

Proteomics of plasma samples before and after the dietary intervention were done using the SomaScan v4.1 assay (SomaLogic) that detects approximately 7,000 proteins. Raw data was provided by SomaLogic following standardization, normalization, and calibration, including adaptive normalization by maximum likelihood (ANML), and mapped to UniProt. Protein measurements are provided in relative fluorescent units (RFU). Data was log2 transformed and standardized prior to analyses in the current study.

### Statistical analyses

#### Feature selection.

In the plasma, CSF, dlPFC, and STG, APOE4 proteins were identified using mutual information ^54^ as previously reported ^1,2^. In plasma and CSF, we identified *APOE* ε4-speci c proteins using all *APOE* ε4 carriers and non-carriers to validate our previous findings where *APOE* ε4 proteins were identified only in non-impaired controls ^2^. In the dlPFC and STG, we identified *APOE* ε4 proteins in non-impaired controls. Proteins with a mutual information value >0.1 were selected for machine learning analyses. We confirmed our feature selection method using principal component analysis (PCA). For plasma and CSF, we also performed a second feature selection using mutual information on *APOE* ε4 carriers with AD relative to non-impaired control *APOE* ε4 carriers. Mutual information was calculated in R (v4.4.1) using the package ‘FSelectorRcpp’ ^55^ and PCA plots were made using ‘ggplot2’ ^56^.

#### Machine learning.

We used classification and regression trees (CART) and random forest to test the predictive performance of our identified *APOE* ε4 proteins for differentiating between *APOE* ε4 carriers and non-carriers. The dataset was split into a 70% training and validation set and a 30% withheld (unseen) testing set. Model training and evaluation were done using a 5-fold cross-validation procedure repeated 10 times. Machine learning was done in R (v4.4.1) using the package ‘caret’.

#### Cortical organoid proteomics.

Label-free mass spectrometry proteomic data was analyzed in R (v4.4.1) using ‘limma’ package. The raw data consisted of protein group intensities across biological samples, with sample names encoding both experimental condition (*APOE* ε4 carrier or non-carrier) and time point (four weeks or eight weeks). The protein intensity matrix was filtered to retain proteins with valid quantification in at least 30% of samples. Missing values were imputed per protein by replacing missing entries with the minimum observed intensity for that protein, assuming missingness was due to low abundance below the detection limit. Protein intensities were log2-transformed to stabilize variance. Differential protein abundance between *APOE* ε4 and non-carrier cortical organoid samples was assessed at both four week and eight-week time points using a linear modeling framework with empirical Bayes moderation, implemented in limma. Specific contrasts (*APOE* ε4 vs non-carrier at each time point) were tested. Resulting *p*-values were corrected for multiple testing using the Benjamini- Hochberg method to control the false discovery rate (FDR). Proteins with adjusted p-values (FDR) below 0.05 were considered significantly differentially abundant. Volcano plots were constructed using -log_10_- transformed adjusted p-values on the y-axis. Plots show significance thresholds that were applied at FDR-adjusted p < 0.05. All plots and downstream analyses were performed in R using the ‘ggplot2’ package.

#### Cortical organoid AD pathology.

ELISA data for secreted amyloid-β42/40 and p-tau217 was analyzed at each time point using a two-tailed unpaired t-test. To analyze interactions between genotype and time, a two-way ANOVA was used. All statistical analyses were performed in GraphPad Prism (v10.5.0).

#### TDAD clinical trial proteomics.

Log2 transformed SomaScan assay data was analyzed in R (v4.4.1) using ‘limma’ package. Differential protein abundance between baseline and post-study in *APOE* ε4 carriers and non-carriers was assessed using a linear modeling framework with empirical Bayes moderation, implemented in limma. Resulting *p*-values were corrected for multiple testing using the Benjamini- Hochberg method to control the false discovery rate (FDR). Proteins with adjusted p-values (FDR) below 0.05 were considered significantly differentially abundant. Volcano plots were constructed using -log_10_- transformed adjusted p-values on the y-axis. Plots show significance thresholds that were applied at FDR-adjusted p < 0.05. All plots and downstream analyses were performed in R using the ‘ggplot2’ package.

#### Enrichment analysis.

Enriched biological functions and pathways across APOE4 proteins were assessed using NetworkAnalyst (v3.0) ^57–59^. Protein-protein interactions were identified using a first order network in the International Molecular Exchange Consortium (IMEx) interactome database ^60^ and InnateDB ^61^. Network enrichments for biological processes and pathways were done using the PANTHER classification system ^62^ and Kyoto Encyclopedia of Genes and Genomes (KEGG) database ^63^, respectively. Statistical significance of the enriched networks was determined by a false discovery rate (FDR) of > 0.05. Proteins enriched in the KEGG AD pathway were visualized using KEGG mapper ^64^. Cell type-specific enrichment analyses for immune cells, brain regions, and white matter cells were done using single-cell RNA sequencing data from the Human Protein Atlas (v23, Ensembl v109) ^12–14^. Here, the corresponding protein-coding transcripts per million for each *APOE* ε4 protein was identified. Expression for each cell type was normalized using min-max scaling. Heatmaps were generated to visually represent these enrichments using R (v4.4.1).

## Supplementary Material

This is a list of supplementary files associated with this preprint. Click to download.

- Shvetcovetal.2025SupplementaryTables.xlsx
- Extendeddata.docx

## Figures and Tables

**Methods Table 1.** Therapeutic Diets in Alzheimer’s Disease (TDAD) clinical trial inclusion and exclusion criteria.

**Inclusion Criteria**	1. Diagnosis of AD by NIA-AA criteria ^47^
	2. CDR global score of 0.5 or 1
	3. Agreed cooperation from an appropriate study partner
	4. Speaks English as a primary language
	5. Age 50 to 90
	6. No medication changes within the past 30 days
**Exclusion Criteria**	1. Resides in a nursing home or dementia special care unit, or cannot control diet
	2. A potentially confounding serious medical risk including insulin-requiring diabetes, cancer requiring chemotherapy or radiation within the past five years, or recent cardiac event (e.g. heart attack, angioplasty, etc.) 3. Participating in another clinical trial or using an investigational drug or therapy within 30 days of the screening visit
	4. A history of renal stones
	5. Women with child-bearing capacity who seek to become pregnant
	6. Non-English speakers

**Methods Table 2.** List of N2/B27 media components.

Reagent	Concentration (in 1000ml)	Source
DMEM/F12	478ml	Gibco, 11320033
Neurobasal medium	480ml	Gibco, 21103049
N2 supplement	5ml	Gibco, 17502048
Human insulin	2.5ug/ml	Sigma-Aldrich, I9278–5ML
MEM NEAA	5ml	Gibco, 11140050
Beta-mercaptoethanol	25mM	Gibco, 21985023
B27 (without vitamin A)	10ml	Gibco, 12587010
GlutaMAX supplement	10ml	Gibco, 35050061
Penicillin-streptomycin	100u/ml	Gibco, 15140122
Dosomorphin	1uM	StemCell Technologies, 72102
SB431542	10uM	StemCell Technologies, 72234
CHIR99021	3uM	StemCell Technologies, 72054
Thiazovivin	2uM	StemCell Technologies, 72254

**Methods Table 3.** List of primary and secondary antibodies.

Antibody	Concentration	Source
Rabbit anti-MAP2	1:250	Invitrogen, PA5–17646
Goat anti-IBA1	1:500	Abcam, ab5076
Rabbit anti- GFAP	1:250	Invitrogen, PA5–16291
Rabbit anti- pTau217	1:200	Invitrogen, 44–744
Mouse anti-APP6E10	5 μg/ml	BioLegend, SIG-39320
Donkey anti-goat Cy3	1:100	Jackson Immunoresearch, 705–165-147
Donkey anti-rabbit AlexaFluor 488	1:100	Invitrogen, A21206
Donkey anti-rabbit AlexaFluor 647	1:100	Invitrogen, A31573
Donkey anti-rabbit Cy3	1:100	Jackson Immunoresearch, 711–165-152
Donkey anti-mouse AlexaFluor 555	1:100	Invitrogen, A31570

## Data Availability

The harmonized GNPC data used to generate these findings was provided to Consortium Members in June 2024 and will be made available for public request by the AD Data Initiative by July 1, 2025. Members of the global research community will be able to access the metadata and place a data use request via the AD Discovery Portal (https://discover.alzheimersdata.org/). Access is contingent on adherence to the GNPC Data Use Agreement and the Publication Policies. The AMP-AD Diverse Cohorts study data is available through the AD Knowledge Portal (https://adknowledgeportal.synapse.org/). Researchers who wish to access this controlled dataset are required to submit a Data Use Agreement. More information can be found here: https://adknowledgeportal.synapse.org/Data%20Access.
